# Y-CpG-based semen age prediction: analysis of vasectomized samples and development of an optimized multiplex assay evaluated in independent and mixed samples

**DOI:** 10.1007/s00414-026-03797-y

**Published:** 2026-04-14

**Authors:** Ji Eun Lee, Hwan Young Lee

**Affiliations:** 1 Department of Forensic Medicine, Seoul National University College of Medicine, 103 Daehak-Ro, Jongno-Gu, Seoul, 03080 South Korea; 2 Institute of Forensic and Anthropological Science, Seoul National University College of Medicine, 103 Daehak-Ro, Jongno-Gu, Seoul, 03080 South Korea

**Keywords:** Methylation, Age, Massively parallel sequencing, Semen, Y-chromosome

## Abstract

Age prediction using DNA methylation has become an important tool in forensic genetics, especially for semen samples in sexual assault cases. Our previous research has identified age-associated CpG markers on the Y-chromosome and developed a multiplex assay along with regression models for predicting the age of semen samples. However, the performance of these methods under biologically altered conditions, such as vasectomized semen and mixed body fluid samples, has not been thoroughly investigated. In this study, we analyzed semen samples from vasectomized individuals using a previously developed 10-amplicon Y-CpG assay to examine methylation patterns and age prediction capabilities. The vasectomized samples were categorized based on body fluid identification results, revealing distinct methylation profiles. We observed markedly reduced age prediction accuracy in samples that lacked semen-specific profiles, while those with semen-specific profiles exhibited patterns similar to normal semen. Building on feature selection outcomes from our earlier study, we further developed an optimized five-amplicon multiplex assay and assessed its performance with independent semen samples and semen-vaginal fluid mixtures. In evaluations involving 11 independent semen samples, the stepwise and lasso models produced mean absolute errors (MAEs) of 6.74 and 6.55 years, respectively. The assay also showed consistent performance with semen-vaginal fluid mixtures, with methylation differences below 2% and average age prediction errors under 6 years. Overall, these findings indicate that the five-amplicon assay offers a streamlined and practical approach for predicting semen age in forensic contexts. Additionally, incorporating body fluid identification may enhance the interpretation of age predictions in challenging samples.

**Supplementary information:**

The online version contains supplementary material available at 10.1007/s00414-026-03797-y.

## Introduction

Semen is a highly valuable form of biological evidence in sexual assault investigations. Its presence can indicate that sexual intercourse or related sexual activity has occurred [1], making it crucial in forensic analysis. In actual casework, biological evidence often exists as a mixture rather than as pure semen, typically combined with other body fluids such as vaginal fluid or blood [2]. In these instances, Y chromosome–specific analysis enables the selective profiling of male DNA, minimizing interference from female DNA. One of the most widely used Y-chromosome-based approaches is Y-short tandem repeat (Y-STR) profiling, which is highly effective for identifying males [3]. However, since Y-STR haplotypes are shared among patrilineal relatives, this method cannot differentiate between individuals such as fathers and sons. Rapidly mutating Y-STRs (RM Y-STRs) have been proposed to address this limitation [4], although their resolution is still limited in certain cases [5]. Therefore, in the context of Y chromosome–based analysis of mixtures, there is a clear need for tools that can distinguish individuals who share identical Y-STR haplotypes, particularly among close paternal relatives. Age prediction represents a promising solution to address this challenge.

DNA methylation is an epigenetic modification that primarily occurs at CpG dinucleotides [6], exhibiting characteristic changes with chronological age [7–9]. It has been established as a reliable biomarker for age prediction, with multiple studies demonstrating that age can be accurately predicted using methylation-based approaches [10–12]. Methylation-based epigenetic clocks have been extensively investigated and progressively improved [11, 13–16]. Additionally, both tissue-combined and tissue-specific age prediction models have been developed [12, 17–23], with subsequent advancements including semen or sperm-specific prediction models [24–32], highlighting their forensic relevance.

Vidaki et al. first introduced age prediction based on Y-chromosomal CpGs using blood samples [33]. Following this, Lee et al. expanded the approach to semen, developing a multiplex assay targeting ten age-associated CpGs that successfully generated methylation data [34]. Feature selection analyses were conducted to identify the markers contributing most to age prediction. Among the evaluated regression methods, the stepwise and Lasso models achieved the highest prediction accuracy, selecting five and seven CpGs, respectively. The stepwise model selected cg15810474, cg14446584, cg00061679, Y:9400045, and Y:23168085, while the Lasso model additionally included Y:9400019 and cg13372258. Notably, all seven CpGs were located within the same five amplicons of the original 10-amplicon multiplex assay. This finding suggests that a simplified multiplex assay focusing on these five amplicons could retain the predictive power of both models while reducing the number of required amplicons.

From a practical forensic perspective, reducing the number of targets is advantageous for low-template or degraded DNA [35]. Larger panels with numerous markers tend to result in higher rates of missing data [36, 37]. An optimized, smaller multiplex can reduce amplification competition, minimizing the occurrence of incomplete or uninterpretable results in challenging forensic samples [38, 39]. In addition, because biological evidence in real casework often exists as mixtures of different bodyfluids [2], it is important to assess performance under such conditions. Therefore, the optimized five-amplicon assay was developed and tested not only with pure semen but also with vaginal fluid–semen mixtures to evaluate its robustness.

Furthermore, semen samples with altered cellular composition, such as those obtained after a vasectomy, provide additional insight into Y-CpG-based age prediction. The absence or significant reduction of spermatozoa may change methylation patterns and affect the performance of age prediction models. In sperm-absent samples, the models that performed best in sperm‑containing semen samples may not remain optimal. This necessitates the application of a broader set of predictive models and the use of the full 10-amplicon assay for evaluation.

This study developed and validated an optimized five-amplicon multiplex assay that targets only the CpGs selected by the final regression models from previous work. Its performance was assessed using independent semen samples and semen–vaginal fluid mixtures to evaluate accuracy and robustness. Additionally, vasectomized semen samples were analyzed using the earlier 10-amplicon assay.

## Methods

### Samples

Semen and vaginal fluid specimens were collected from healthy Korean volunteers with appropriate Institutional Review Board (IRB) approvals. Written informed consent was obtained from all participants prior to sample collection. This study included 16 normal semen samples, 16 vasectomized semen samples, and 6 vaginal fluid samples, all approved by Yonsei University Severance Hospital and Seoul National University Hospital (IRB Nos. 4–2015-0083 and 1912–053–1087). Semen specimens were collected in sterile plastic cups, aliquoted into 1.5 mL microtubes, and stored at −80 ℃. Vaginal fluid samples were collected using sterile cotton swabs, air-dried, placed in paper envelopes, and then preserved at −80 ℃.

### DNA extraction and bisulfite conversion

DNA extraction was performed using the QIAamp DNA Mini Kit (Qiagen) following the manufacturer’s instructions. Dithiothreitol (DTT) was added to the semen samples as described in [34]. The extracted DNA was stored at −20 ℃, and DNA quantification was conducted using the Quantifiler Trio DNA Quantification Kit (Applied Biosystems, Foster City, CA, USA).

For mixture preparation, semen and vaginal fluid DNA were mixed based on Quantifiler Trio measurements, with semen DNA quantified using the Y-chromosome target and vaginal fluid DNA quantified using the Large autosomal target. This quantification strategy aligns with the assay’s focus on Y-chromosomal CpG markers for age prediction. A total of 35 ng of semen DNA was mixed with 350 ng, 525 ng, or 700 ng of vaginal fluid DNA, corresponding to ratios of 1:10, 1:15, and 1:20, respectively. For each ratio, three independent mixtures were prepared using different samples, resulting in a total of nine mixtures. In total, semen DNA from four normal samples collected in 2015 and five samples collected in 2022 was used, while vaginal fluid DNA was obtained from six samples collected in 2022, with three samples used twice across the mixtures. To ensure age diversity, the semen donors included two in their 20 s, two in their 30 s, one in his 40 s, two in their 50 s, and two in their 60 s. Vaginal fluid donors included one in her 20 s, one in her 30 s, one in her 40 s, two in their 50 s, and one in her 60 s.

For bisulfite conversion, 100 ng of semen DNA from single-origin samples was used as input, and the DNA was eluted in 10 μL. For mixture samples, the input was adjusted to contain 25 ng of semen DNA, and also eluted in 10 μL, resulting in a converted DNA concentration of approximately 2.5 ng/μL. Bisulfite conversion was performed using the EZ DNA Methylation-Lightning Kit (Zymo Research, Irvine, CA, USA), and all converted DNA was stored at − 80 °C until use.

### Body fluid identification

All samples included in this study were verified using the body fluid identification assay suggested by Lee et al. [40]. Experiments followed the original protocol, utilizing converted DNA at an input concentration of 10 ng/μL. The purified SBE products were analyzed with a 3500 Genetic Analyzer (Applied Biosystems, Foster City, CA, USA), and the resulting SNaPshot data were interpreted using GeneMapper Software 5.

### Massively parallel sequencing (MPS)

#### Development of the optimized 5-amplicon multiplex assay

A new multiplex assay was developed, comprising 10 primers targeting 5 amplicons. This 5-amplicon assay was specifically designed to include only the amplicons used in the selected age prediction models. The primer sequences were identical to those used in the original 10-amplicon assay (Supplementary Table 1). However, the primer concentrations were adjusted in the 5-amplicon assay to achieve balanced peak heights across the amplicons, as detailed in Table 1, with representative peak profiles shown in Supplementary Fig. 1. Except for the amount of PCR input, all conditions and experimental steps in the MPS workflow remained the same as those reported in the original study [34].

**Table 1:** Primer concentrations for the optimized 5-amplicon multiplex assay. The genomic locations of the target sites are indicated based on the GRCh38 reference genome, and the amplicon numbers correspond to those of the original study

Amplicon	Target site (Y)	Final Conc. (uM)
2	9400045, 9400019	0.4
3	9468841	0.35
6	10093006	0.7
7	12710685	2.2
10	23168025, 23168085	4.0

#### Library preparation and sequencing

Library preparation involved two consecutive PCR reactions. For single‑source samples, the first‑round PCR was performed in a 20 μL reaction consisting of 10 μL of Qiagen Multiplex PCR Master Mix, 4 μL of a pooled primer mixture, 5 μL of distilled water, and 1 μL of bisulfite converted DNA. For amplification of mixture samples, the volume of converted DNA added to the reaction was increased to 4 μL (approximately 10 ng of semen-derived DNA), and the amount of distilled water was reduced accordingly to maintain a final reaction volume of 20 μL. All reactions were conducted on a Veriti™ 96‑well thermal cycler with the following thermal conditions: 95 °C for 15 min; 34 cycles of 94 °C for 30 s, 57 °C for 90 s, and 72 °C for 90 s; followed by a final extension step at 72 °C for 10 min. For vasectomized samples, the previously developed 10-amplicon multiplex assay was applied, allowing for a more comprehensive comparison with normal semen samples. A second PCR was performed to attach the dual indexes and sequencing adapters. This reaction also utilized a 20 μL total volume, comprising 10 μL of Qiagen Multiplex PCR Master Mix, 2 μL each of the N and S index components from the Nextera XT index kit (Illumina), 5 μL of distilled water, and 1 μL of the diluted first‑round PCR product. The indexing PCR was conducted under the following conditions: 95 °C for 15 min; 17 cycles of 94 °C for 20 s, 61 °C for 30 s, and 72 °C for 90 s; followed by a final extension step at 72 °C of 5 min. Each multiplex PCR amplicon was quantified using a 2100 Bioanalyzer, adjusted to 10 ng in a final volume of 5 μL, and combined to create a pooled library. The pooled mixture was purified using AMPure XP beads (Beckman Coulter, Brea, CA, USA) following the SPRIselect protocol, with right‑side size selection performed at 0.5 × and 1.1 × bead ratios. After purification, the pooled library was quantified using the KAPA Library Quantification Kit (Roche, Basel, Switzerland) according to the manufacturer’s instructions. The purified library was then adjusted to a final concentration of 30 nM prior to sequencing. Paired-end massively parallel sequencing (MPS) was performed on an Illumina MiSeq platform with a 2 × 300 bp configuration by Macrogen Inc. (Seoul, Republic of Korea).

### Data analysis

Raw MPS data were analyzed using the CLC Genomics Workbench 21.0.5 (Qiagen) according to the manufacturer’s instructions, with reads aligned to the hg38 reference genome. Before conducting methylation analysis, a coverage threshold of 200 reads was applied, in line with the criteria described in the original study [34].

Methylation levels in vasectomized semen samples were analyzed using a 10-amplicon assay and compared to the normal sample data reported in the original study [34]. Although the assay included ten amplicons, one was excluded from the analysis due to technical issues reported in the original study. The remaining nine amplicons contributed 32 CpGs for subsequent analyses. To assess methylation differences independent of age, age-adjusted residuals were calculated for each CpG site by fitting linear regression models of methylation on age. Linear regression analyses were conducted in Python using the statsmodels package [41]. Residuals were defined as the difference between observed methylation levels and those predicted based solely on age, with positive values indicating higher methylation than expected and negative values indicating lower methylation. Principal component analysis (PCA) was performed to explore the overall patterns of variation in methylation profiles across sample groups. The PCA was conducted in Python using the scikit-learn package [42], with methylation values standardized prior to analysis. Data visualizations were generated using the matplotlib library [43].

Age prediction was conducted using regression models developed in the original study [34]. No additional model training, optimization, or modification was performed in the current study, and all age predictions relied on the original regression coefficients. Vasectomized semen samples were analyzed using the original 10-amplicon assay, which includes the complete set of 32 CpG markers used for model construction. Consequently, age predictions were conducted using 4 models, including the multiple linear regression (MLR), stepwise, ridge, and lasso regression. Normal semen samples and semen–vaginal fluid mixture samples were analyzed with the optimized 5-amplicon assay, which comprises a total of 18 CpGs. Age prediction for these samples utilized the stepwise and lasso regression models based on the 7 CpGs included in the optimized assay.

To identify epimutated outlier methylation values, we calculated interquartile range (IQR) based thresholds for seven target CpG markers using data from 147 semen samples analyzed in the original study. The first quartile (Q1) represents the 25th percentile, while the third quartile (Q3) represents the 75th percentile of the methylation values. Outliers were defined as values below Q1–3 × IQR or above Q3 + 3 × IQR, where IQR = Q3 – Q1 [44].

Mixture data were analyzed in two ways: first, by examining the differences in methylation values, and second, by assessing the differences in predicted ages. For each mixture, we compared the methylation values and predicted ages to those obtained from the corresponding single-source semen samples.

## Results

### MPS results

Approximately 4.9 million reads were generated, resulting in about 1.1 billion bp of sequencing data. On average, 78.9% of the data showed a quality score above Q30. Sequencing coverage per sample ranged from 59,390 to 188,655 reads, while the coverage per marker within individual samples ranged from 339 to 20,106 reads.

### Vasectomized sample results

In this study, we analyzed 16 vasectomized semen samples and classified them into two subgroups based on their BFI results. Four samples showed clear semen-specific BFI profiles and were designated as Vasec_BFI +, while the remaining 12 samples presented inconclusive BFI results without semen-specific profiles and were designated as Vasec_BFI-. Representative electropherograms of these BFI results are shown in Supplementary Fig. 2.

**Fig. 1:**
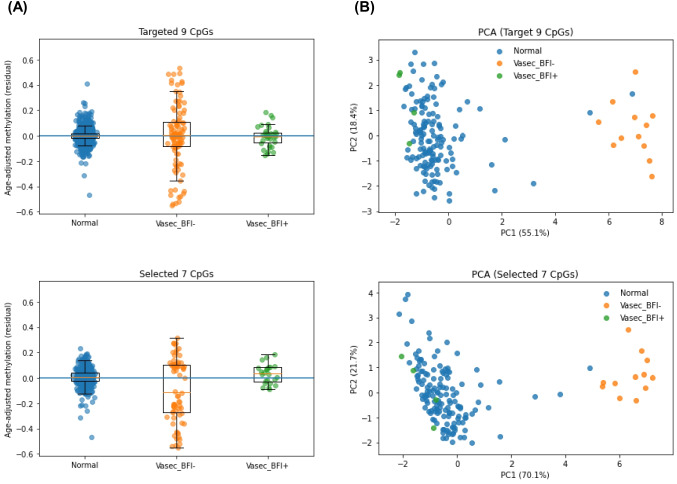
Methylation differences in vasectomized semen samples after accounting for age. **A** Distribution of methylation residuals adjusted for age across sample groups. Residuals were obtained from regression analysis of methylation levels on age for each CpG site, with positive and negative values indicating higher or lower methylation than expected for a given age, respectively. The horizontal line at 0 represents the methylation level predicted based on age. Each dot corresponds to a residual value from an individual CpG site, and boxplots summarize the distribution within each sample group. The top panel shows results for 9 CpGs that comprise the 10-amplicon assay, and the bottom panel shows the 7 CpGs selected for age prediction models. **B** PCA of methylation profiles across sample groups. PCA was performed using methylation levels for all 32 CpGs (top) and the 7 selected CpGs (bottom). Each point represents an individual sample, colored by sample group. The percentage of variance explained by each principal component is indicated on the axes

To assess the methylation characteristics of the vasectomized semen samples, we analyzed methylation levels using a previously developed 10-amplicon assay and compared these levels to data from 147 normal semen samples analyzed in the original study. We examined age-adjusted methylation residuals across the sample groups (Fig. 1, Supplementary Table 2) to evaluate differences independent of age. The residual distributions indicated that Vasec_BFI − samples showed a systematic shift away from zero compared to normal semen samples, suggesting altered methylation levels beyond what would be expected based solely on age. This pattern was consistently observed for both 9 CpGs in the 10-amplicon assay and the 7 CpGs used in age prediction models. In contrast, Vasec_BFI + samples largely overlapped with normal semen samples, showing only minor deviations in specific CpG sets.

To further investigate the overall variation in methylation profiles, we conducted a PCA (Fig. 1). The PCA results indicated that Vasec_BFI − samples tended to cluster separately from normal semen samples, while Vasec_BFI + samples were predominantly found within the normal sample cluster. Some partial overlap between Vasec_BFI − and normal samples was observed, reflecting the inter-individual variability in methylation patterns.

Age prediction for vasectomized semen samples was conducted using four regression models, including MLR, stepwise, ridge and lasso (Supplementary Fig. 3). For the Vasec_BFI + samples, the age prediction errors were relatively low, with the mean absolute error (MAE) values of 5.4 years for the MLR, 3.9 years for the stepwise, 6.4 years for the ridge, and 4.5 years for the lasso model. In contrast, for the Vasec_BFI- samples, the MAE values were markedly higher: 36.9 years for the MLR, 23.0 years for the stepwise, 36.1 years for the ridge, and 25.1 years for the lasso regression models. Notably, for one Vasec_BFI − sample, the MLR model produced a negative predicted age.

### Age prediction results

**Fig. 2:**
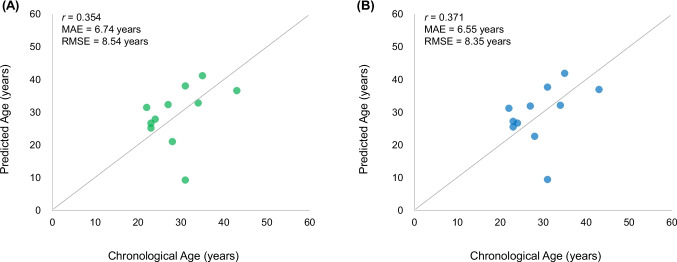
Age prediction results of 11 independent semen samples using the optimized 5-amplicon assay. Each point represents an individual sample, with the x-axis representing chronological age and the y-axis representing predicted age. **A** Results of the stepwise model. **B** Results of the lasso model

When the two recommended models from the original study [34] were applied to 11 semen samples, the MAEs were 6.74 years for the stepwise model and 6.55 years for the lasso model. The corresponding root mean square error (RMSE) values were 8.54 years and 8.35 years, respectively. The age prediction results are presented in Fig. 2. However, one sample showed markedly deviated predicted ages. The donor was 31 years old, but the predicted ages were 9.3 and 9.5 years for the stepwise and lasso models, respectively. After excluding this outlier, the MAEs were decreased to 5.24 and 5.05 years. The age prediction graphs without this sample can be found in Supplementary Fig. 4.

To further investigate this sample, we compared methylation values with IQR-based thresholds derived from the original study’s data [34]. For CpG sites with methylation values close to zero, the lower IQR-based outlier threshold (Q1 − 3 × IQR) extended below zero. This reflects the statistical definition of the threshold and does not imply negative methylation values. Three CpG markers showed methylation levels outside the IQR, with values intermediate between those of normal and vasectomized semen samples (Supplementary Fig. 5). Additionally, this sample generated a semen-specific peak in the body fluid identification assay; however, the heights of the methylation peaks were significantly lower than those observed in other normal samples (indicated by a red arrow in Supplementary Fig. 6 A).

### Mixture data

When mixture data were compared with the corresponding single-source semen data, differences in methylation values and predicted ages were assessed. For all 18 CpG sites, the mean absolute methylation differences were 0.015 ± 0.015, 0.015 ± 0.016, and 0.016 ± 0.013 for 10:1, 15:1, and 20:1 mixtures, respectively (Supplementary Fig. 7 A). For the 7 target CpGs, the differences were 0.014 ± 0.017, 0.016 ± 0.017, and 0.015 ± 0.012, respectively (Supplementary Fig. 7B). In terms of predicted ages, the stepwise model produced mean absolute differences to single-source semen of 3.65 ± 2.26 years, 4.11 ± 2.25 years, and 5.13 ± 3.11 years for 10:1, 15:1, and 20:1 mixtures, respectively (Supplementary Table 3). For the lasso model, the differences were 2.31 ± 2.12 years, 3.75 ± 1.75 years, and 4.05 ± 2.78 years for the same ratios (Supplementary Table 3).

## Discussion

In this study, we developed an optimized multiplex assay for predicting semen age based on Y-CpG markers and validated it using independent semen samples, as well as mixtures of semen and vaginal fluid. Additionally, we analyzed semen samples from vasectomized males to evaluate the age prediction outcomes in this specific group.

The newly developed 5-amplicon multiplex assay was validated using 11 semen samples that were completely independent of those used in the original study [34]. Both the stepwise and lasso models showed MAE values below 7 years, which were slightly higher than the MAE of 5.87 years reported in the original study. Excluding one outlier sample further improved the predictive accuracy, supporting the robustness of the optimized assay and its comparable performance to the original 10-amplicon assay. The abnormal methylation patterns observed in this outlier sample suggest a potential sperm-related issue, underscoring the importance of identifying outliers prior to age prediction. Each marker has a characteristic range of normal methylation values, and substantial deviations from this range can impair prediction accuracy. The portal provided by the Department of Forensic Medicine at Seoul National University College of Medicine (https://forensicdna.snu.ac.kr/portal) enables the visualization of input methylation values within the overall distribution of the reference dataset. By confirming whether a sample represents a marked outlier before age prediction, the reliability of the models in forensic casework can be improved. Additionally, since the age prediction models were developed exclusively using adult semen samples, predicted ages below 18 years may indicate that a sample falls outside the intended scope of the models. For unknown forensic samples yielding such results, predictions should be interpreted with caution, as the models were not developed or validated for individuals under 18 years of age.

A gene-level inspection of the three out-of-range markers was also conducted. Among them, cg13372258 is not annotated to any known gene in the array manifest, whereas cg14446584 maps to *USP9Y* and the Y:23168085 amplicon targets *DAZ1*. Both *USP9Y* and *DAZ1* have been reported to play roles in spermatogenesis [45, 46]. Deviations in methylation levels near such loci could reflect altered sperm composition or impaired spermatogenic function, which is consistent with the intermediate methylation pattern between normal and vasectomized semen, as well as the reduced methylation peak heights observed in this sample. While clinical data were not available for confirmation, these combined signals support the interpretation that the outlier likely arose from an underlying sperm-related issue rather than a systematic bias of the age prediction model.

In addition, we further analyzed vasectomized semen samples using the previously developed 10-amplicon assay to assess their age prediction performance in relation to BFI status. The methylation patterns and age prediction accuracy in these vasectomized samples varied markedly based on the presence of semen-specific BFI profiles. After accounting for age, Vasec_BFI − samples exhibited systematic deviations in methylation residuals compared to normal semen samples, whereas Vasec_BFI + samples showed patterns comparable to those of normal semen samples. This distinction was consistently supported by PCA, which indicated that Vasec_BFI − samples tended to occupy a region distinct from normal semen samples, while Vasec_BFI + samples largely overlapped with the normal sample distribution. There was some partial overlap between Vasec_BFI − and normal samples, reflecting inter-individual variability and the heterogeneous contribution of CpG markers, with some CpGs showing large methylation differences and others exhibiting only minimal differences between the groups.

These methylation differences were reflected in the performance of age prediction models. When four age prediction models developed in the original study were applied to vasectomized semen samples, the Vasec_BFI − samples exhibited markedly reduced prediction accuracy, with large MAE values across all models. In contrast, the Vasec_BFI + samples yielded substantially lower errors, particularly in the stepwise and lasso models. This discrepancy is consistent with the fact that these models were developed using DNA derived from semen containing intact sperm cells, whereas vasectomized samples may lack spermatozoa or contain altered cellular compositions. Importantly, vasectomy status in our cohort was self-reported rather than clinically verified. Therefore, misclassification cannot be excluded, and rare occurrences of primary failure or post-operative recanalization could allow spermatozoa to persist or reappear in the ejaculate [47, 48]. Overall, these findings indicate that BFI results provide important contextual information for interpreting methylation-based age marker data in vasectomized semen samples. They also suggest that incorporating a BFI test may enhance the reliability of age predictions in semen.

For mixture analysis, three ratios were examined to simulate conditions where the female proportion is relatively high, making it difficult to clearly obtain the male profile. STR markers, commonly used for DNA profiling, often generate stutter products (N ± 1 repeats) during PCR amplification [49]. Because of this artifact, traditional STR-CE analysis typically produces reliable profiles only when the minor contributor constitutes more than 5–10% of the DNA mixture, making it less suitable for severely unbalanced samples [50]. Given this limitation, the mixture experiments in the present study were designed starting with a 10:1 ratio.

To further verify the applicability of the models, semen–vaginal fluid mixtures were prepared at three ratios (10:1, 15:1, and 20:1) with three independent mixtures for each ratio to ensure a more reliable evaluation. Data were analyzed in terms of both methylation value differences and predicted age differences, each compared with the corresponding single-source semen samples. In the methylation-based approach, values of 18 CpG markers were compared with those from the matched single-source semen data. For each ratio, the mean differences among the three mixtures were below 2% for all CpG markers. This level of variation is comparable to the reproducibility test reported in the original study (mean difference of 1.7%), demonstrating the accurate measurement of the semen component within the mixtures.

In the age prediction approach, the mean absolute differences were approximately 3.7 years for 10:1, 4.1 years for 15:1, and 5.1 years for 20:1 ratio in the stepwise model and 2.3 years for 10:1, 3.8 years for 15:1, and 4.0 years for 20:1 ratio in the lasso model. The average error for both models was less than 6 years. When combined with the additional error of approximately 5 years observed in the 20:1 mixtures, the cumulative prediction error could reach around 11 years. Nevertheless, since the primary aim of this study is to distinguish potential father–son relationships among suspects, such a level of error would not necessarily compromise practical applicability. This level of variability is likely attributable to the limited number of CpG markers included in the models. The regression coefficients of individual markers are relatively large, meaning that even small shifts in methylation values can result in substantial differences in predicted ages.

When examining prediction differences, the predicted ages for semen–vaginal fluid mixtures tended to be higher than those obtained from the corresponding single-source semen samples. One possible explanation is low-level signals that may arise from technical factors. Due to the small size of PCR amplicons and the sequence simplification caused by bisulfite conversion, the specificity of primer binding may be reduced, increasing the likelihood of non-specific amplification and potential primer mispairing to non-target regions. In the original study [34], analysis of female reference samples showed that several CpG markers exhibited low but non-zero methylation levels, even though substantially lower coverage compared to semen samples. Notably, some of these CpGs overlap with the seven markers used for age prediction. Consequently, in semen–vaginal fluid mixtures, such factors may contribute to the observed shift in predicted age. In addition, only nine samples were analyzed without technical replicates, and the relatively low coverage threshold of 200 reads may further contribute to stochastic variation between measurements. Importantly, this effect did not result in extreme prediction errors, suggesting that the influence is a systematic but relatively moderate, rather than a substantially compromising predictive reliability.

In routine forensic casework, differential extraction is commonly used to separate sperm DNA from mixtures of semen and vaginal fluid. However, this method is not always successful or applicable, particularly when samples are limited in quantity or degraded. For this reason, the original assay was designed to analyze samples without differential extraction. In the present study, mixture samples were similarly analyzed without differential extraction, following the experimental design of the original assay. The original study examined the effect of epithelial and round cell admixture on age prediction accuracy, and the results indicated that variations in the proportion of sperm cells in semen samples did not substantially affect age prediction performance. These findings suggest that the model may be applicable regardless of whether differential extraction is used.

In practical forensic applications, these findings suggest that age prediction could also be feasible in sexual assault casework if sufficient amounts of male DNA are recovered. Specifically, reliable age estimates are likely to be obtained when the Y-chromosomal DNA quantified from evidentiary samples exceeds approximately 10 ng. The mixtures in this study were prepared based on DNA concentrations measured with the Quantifiler Trio kit, however, the resulting ratios may not precisely match the intended proportions. This discrepancy arises because real-time PCR–based quantification is highly sensitive, and even minor technical variations can influence measured concentrations, leading to deviations from the expected ratios [51]. Supplementary Table 4 presents the quantification results for the mixtures used in this study, providing a reference for the ratios that can support robust age prediction.

## Conclusion

In this study, we developed a five-amplicon multiplex assay for predicting semen age based on Y-CpG methylation and evaluated its performance using independent semen samples and semen–vaginal fluid mixtures. The assay demonstrated predictive accuracy comparable to that of the previously developed 10-amplicon assay, with mean absolute errors of less than 7 years in the independent semen samples. Additionally, consistent performance was also observed in the semen–vaginal fluid mixtures, reflecting realistic forensic scenarios. Semen samples from vasectomized individuals were also analyzed using the original 10-amplicon assay to examine methylation patterns and age prediction performance. The vasectomized samples showed substantially larger prediction errors, while reasonably accurate predictions were obtained when semen-specific body fluid identification results were available. These findings demonstrate the forensic utility of the current assay and suggest that integrating body fluid identification could further improve the reliability of age prediction in challenging casework samples.

## Supplementary information

Below is the link to the electronic supplementary material.

Supplementary file1 (PDF 694 kb)

Supplementary file2 (XLSX 20 kb)

## Data Availability

The data that support the findings of this study are available from the corresponding author upon reasonable request.
