# Supplementary material for: Y-CpG-based semen age prediction: analysis of vasectomized samples and development of an optimized multiplex assay evaluated in independent and mixed samples
Source: Int J Legal Med. 2026 Apr 14;140(4):2055–64. doi: 10.1007/s00414-026-03797-y (PMC13275618; doi:10.1007/s00414-026-03797-y)

Supplementary Figure 1. Representative electropherogram showing balanced peak heights across the five amplicons in the optimized 5plex multiplex assay. PCR products were generated using FAM-labeled primers and analyzed by capillary electrophoresis on an ABI 3500 Genetic Analyzer. Fluorescent signals were detected and visualized as peaks. Peak heights (RFU) are shown on the y-axis and fragment sizes (bp) on the x-axis. Peaks correspond to amplicons 2, 3, 7, 6, and 10 (from left to right), indicating balanced amplification across all targets.

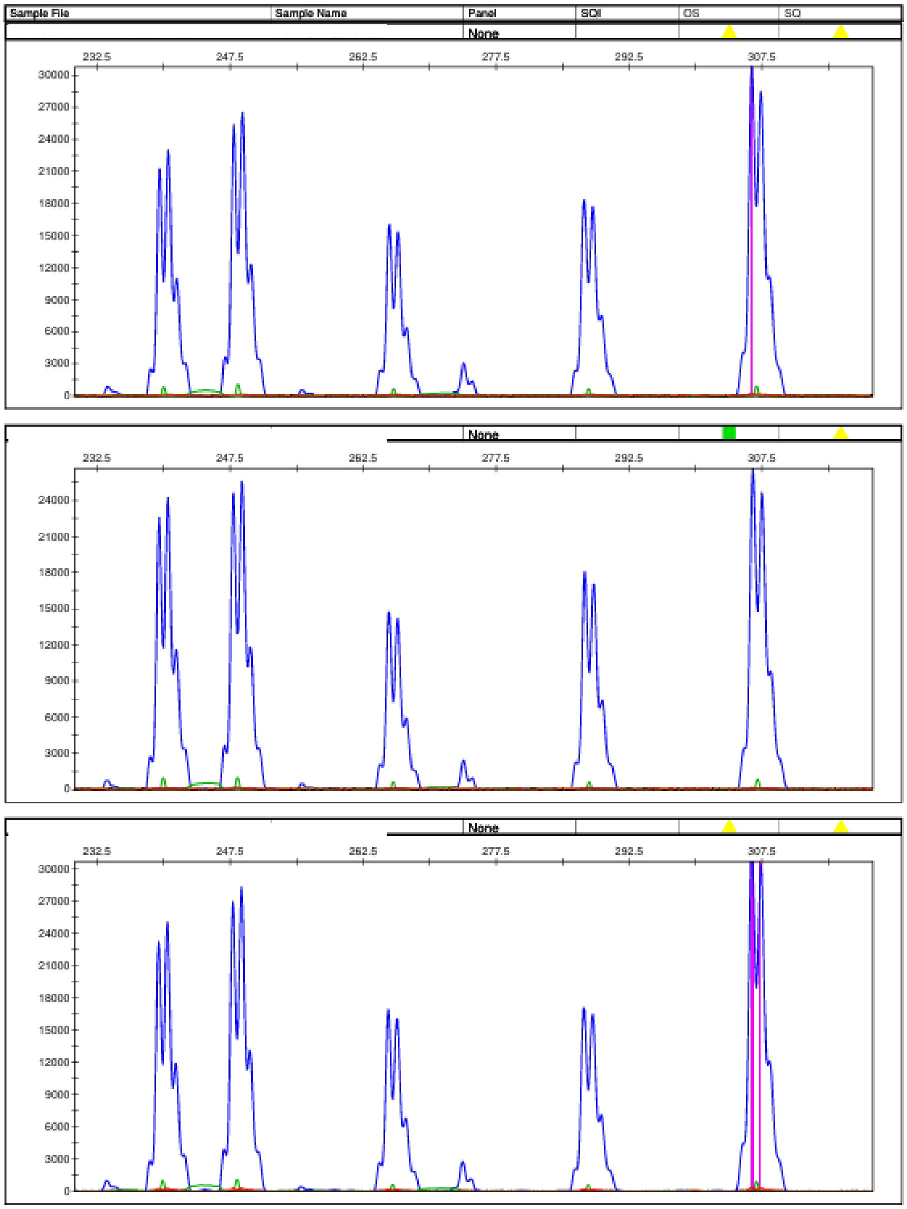

Supplementary Figure 2. Bodyfluid identification results of vasectomized semen samples. 9 markers for semen (SE), blood (BL), vaginal fluid (VF), saliva (SA) and menstrual blood (MB) are utilized. 4 samples with typical semen methylation pattern were marked with red arrows.

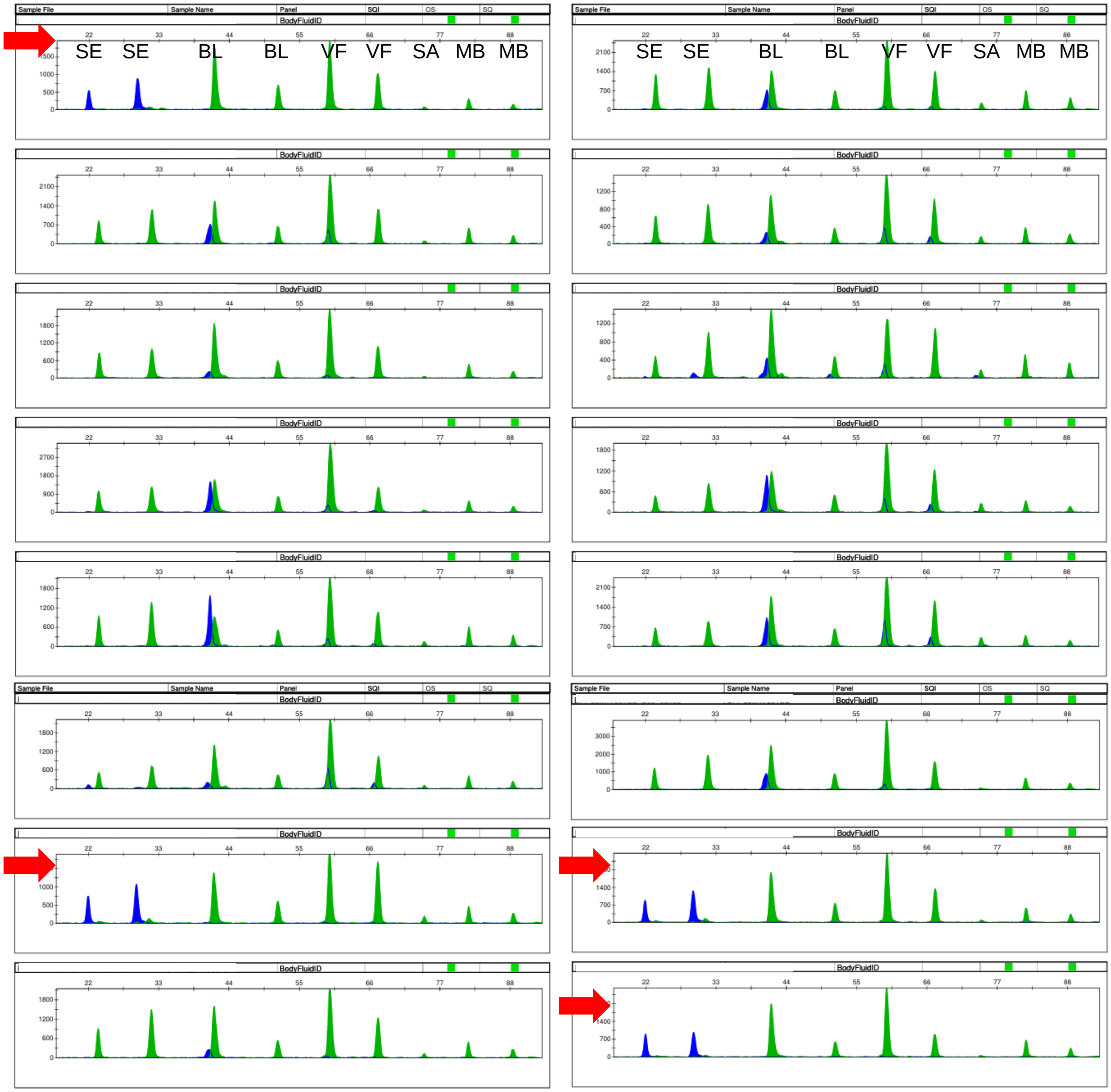

Supplementary Figure 3. Graphs of age prediction results for vasectomized semen samples. Ages were predicted using (A) multiple linear regression model with 9 CpGs, (B) stepwise model with 5 CpGs, (C) ridge model with 32 CpGs, and (D) lasso model with 7 CpGs. Samples exhibiting semen-specific bodyfluid identification profiles are indicated in orange, while samples with inconclusive profiles are indicated in green.

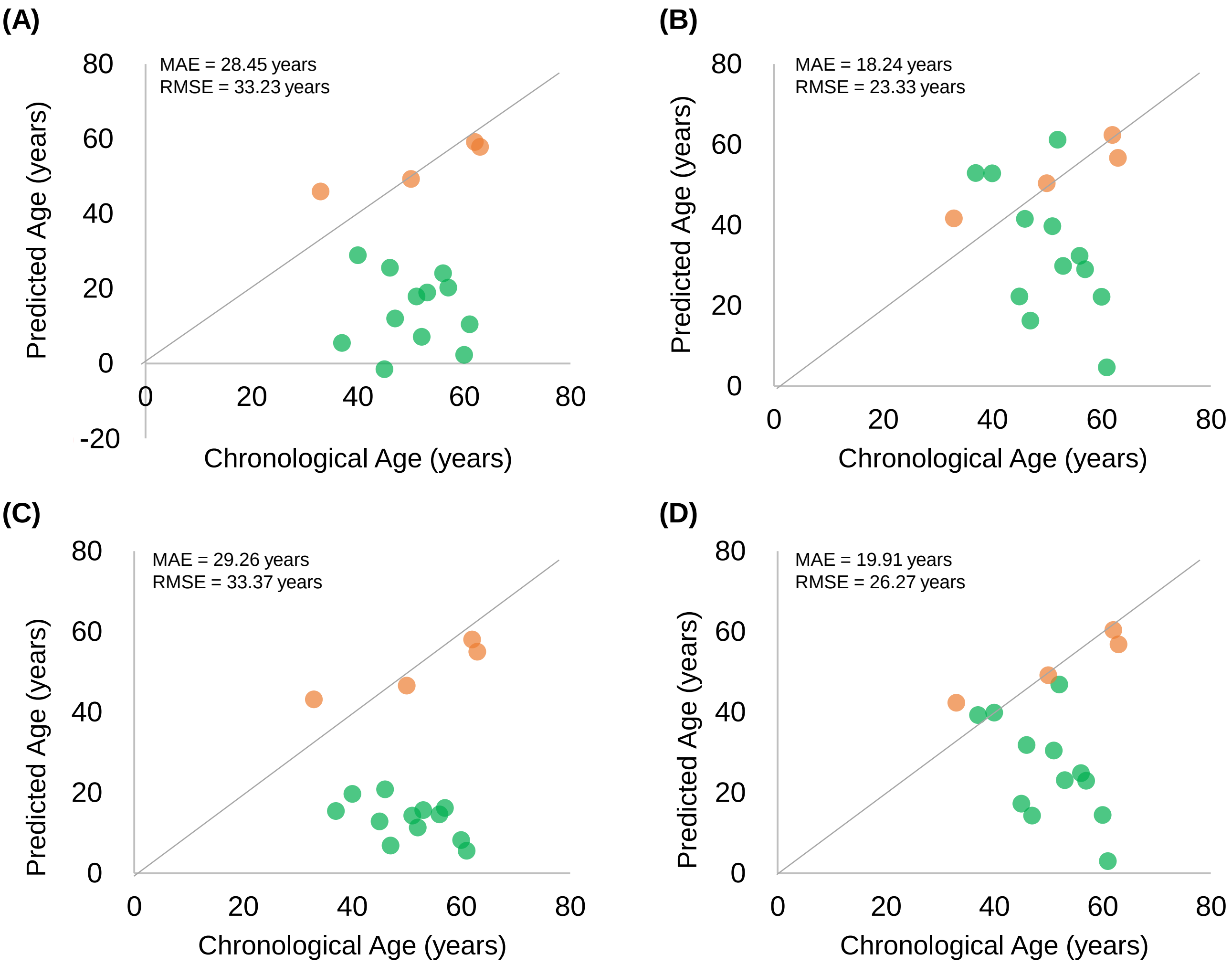

Supplementary Figure 4. Age prediction results of 10 semen samples except the outlier sample. Ages were predicted using (A) stepwise model and (B) lasso model.

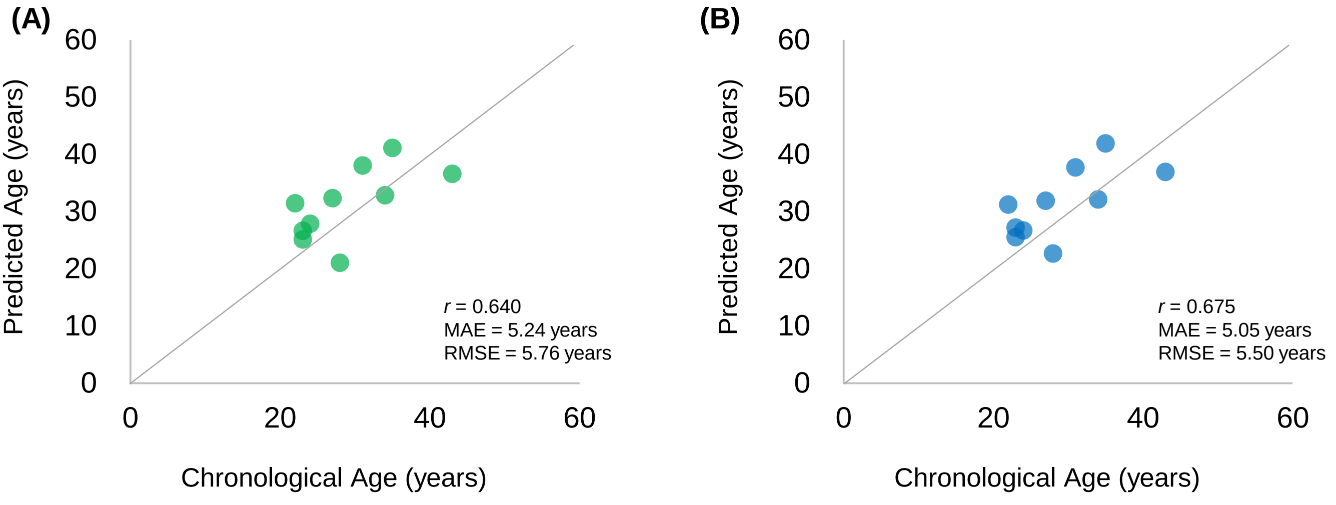

Supplementary Figure 5. Methylation values of the outlier semen sample at CpG sites showing IQR-defined deviations compared with reference data. (A) Bar plots show methylation levels of cg13372258, cg14446584, and Y:23168085, with the outlier sample indicated by pink arrows. (B) The table summarizes mean methylation values (± SD) obtained from the original study data (N = 147) and vasectomized semen samples (N = 12) that did not include samples with semen-specific methylation patterns in the body fluid identification assay.

(A)

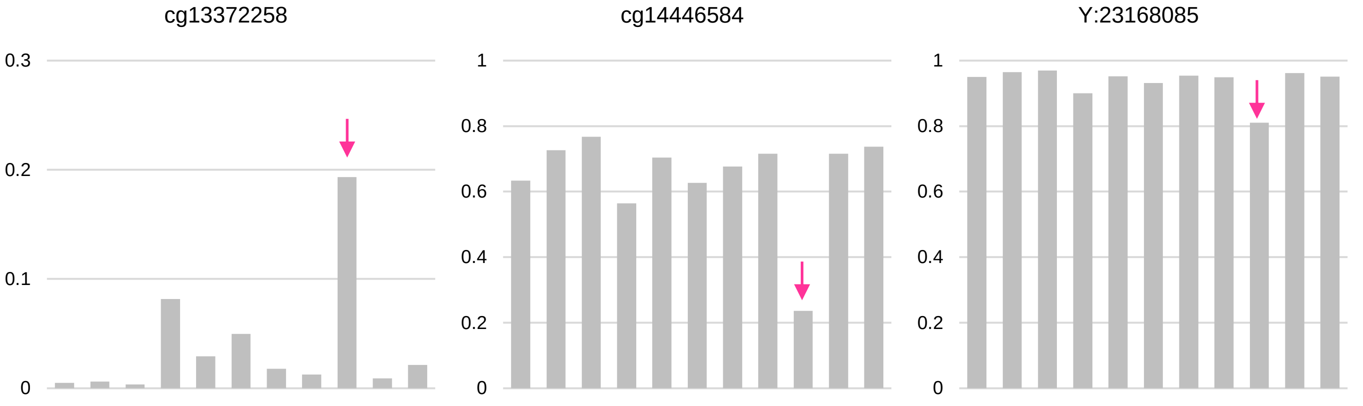

(B)

	**cg13372258**	**cg14446584**	**Y:23168085**
Original study data (N=147)	0.024 ± 0.032	0.73 ± 0.08	0.95 ± 0.03
IQR	-0.04 ~ 0.07	0.42 ~ 1.05	0.92 ~ 0.99
Vasectomized sample data (N=12)	0.30 ± 0.05	0.20 ± 0.06	0.65 ± 0.06
Outlier sample	0.19	0.24	0.81

Supplementary Figure 6. Bodyfluid identification results of (A) semen and (B) vaginal fluid samples collected in 2022. 9 markers for semen (SE), blood (BL), vaginal fluid (VF), saliva (SA) and menstrual blood (MB) are utilized. The result of the outlier sample is marked with a red arrow.

(A)

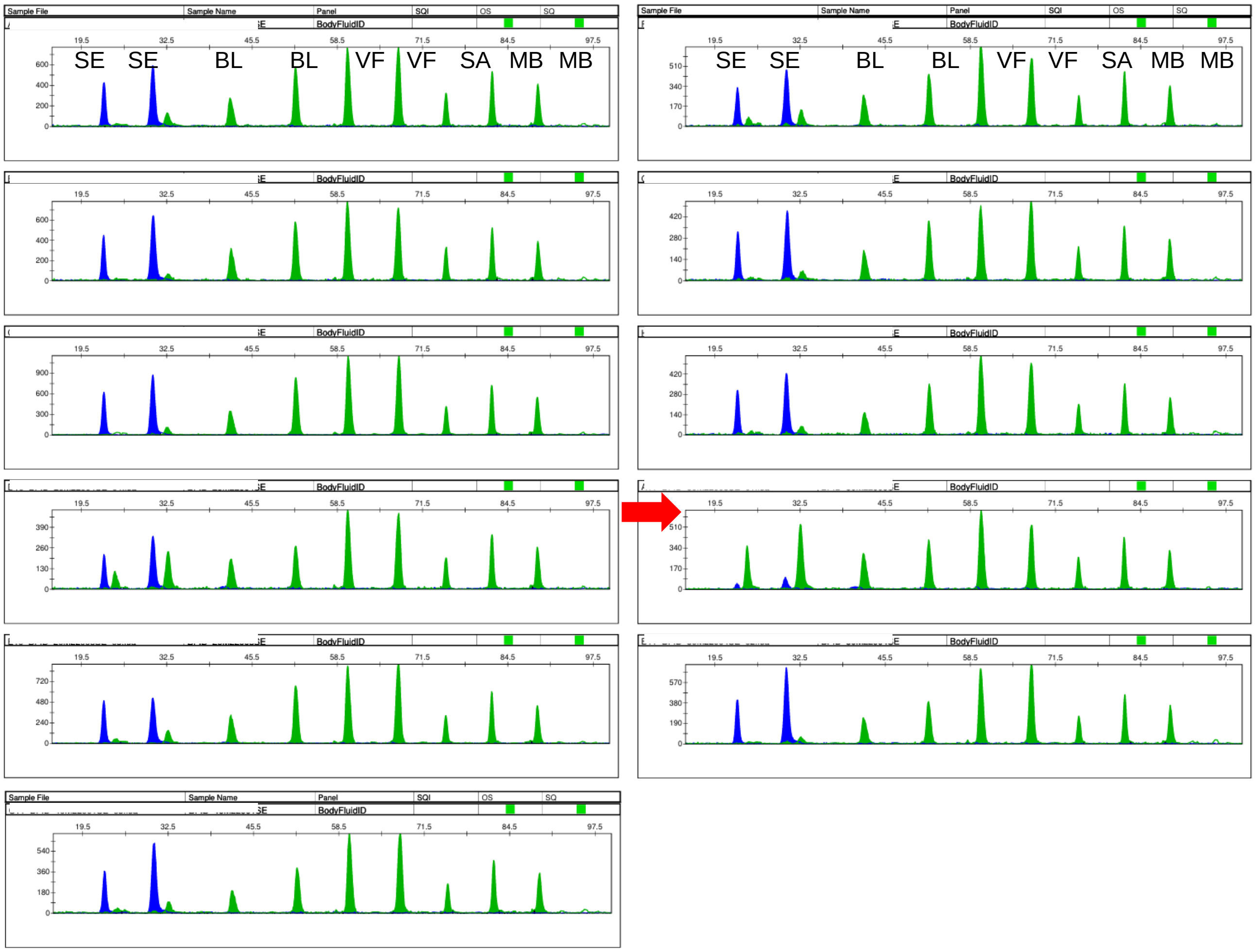

(B)

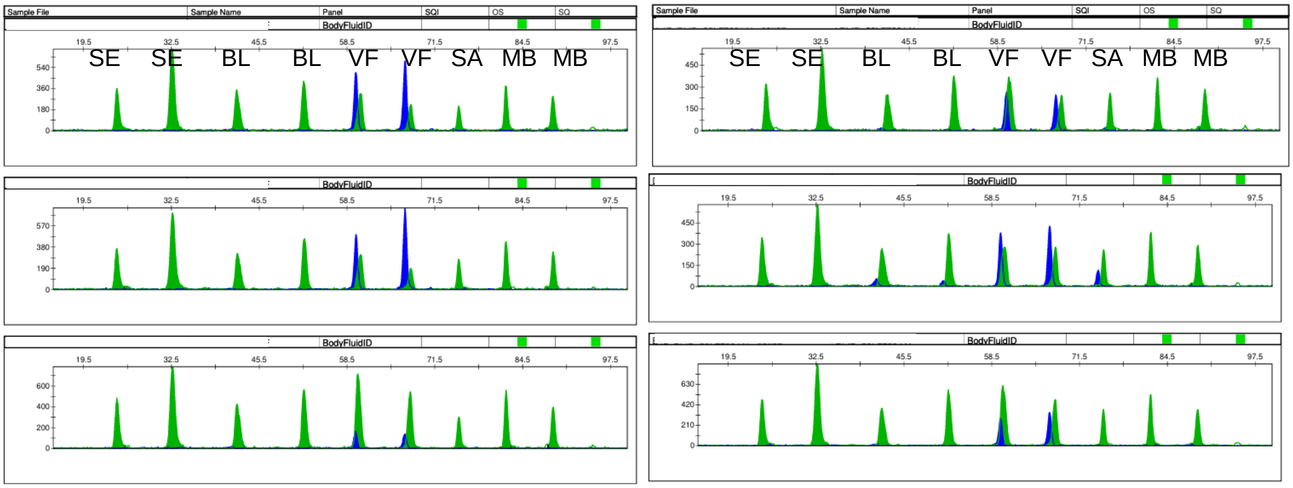

Supplementary Figure 7. Comparison of methylation levels between semen–vaginal fluid mixtures and the corresponding single-source semen samples. The x-axis represents mixture ratios (male:female), and the y-axis represents absolute differences in methylation levels. Boxplots display the distribution of absolute methylation differences for each mixture sample across (A) all 18 CpG markers and (B) targeted 7 CpG markers.

(A)

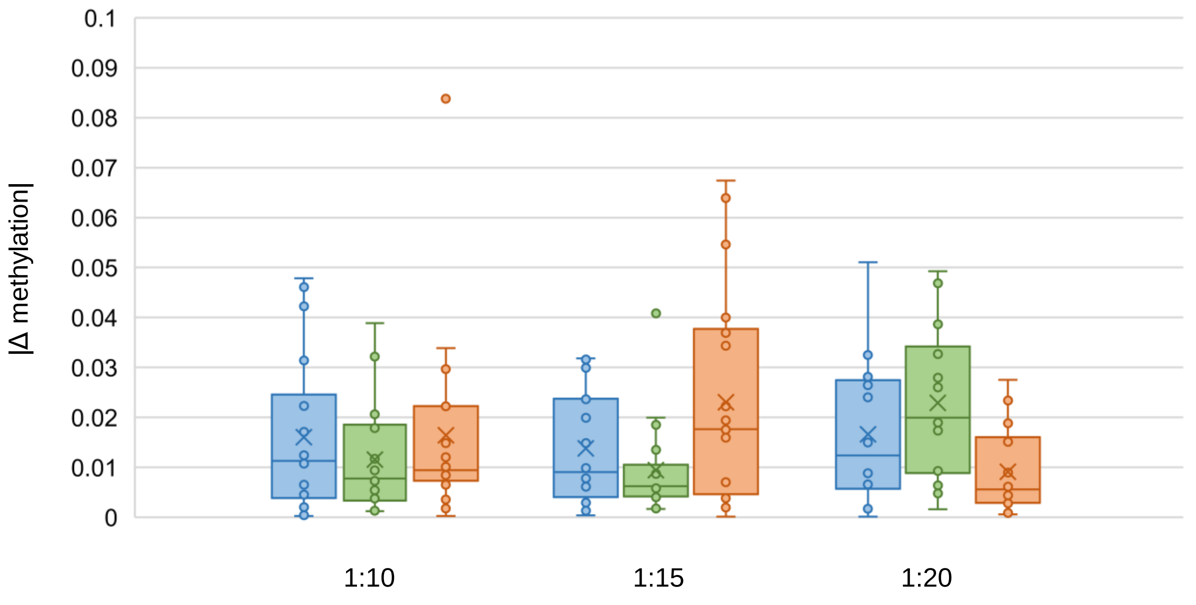

(B)

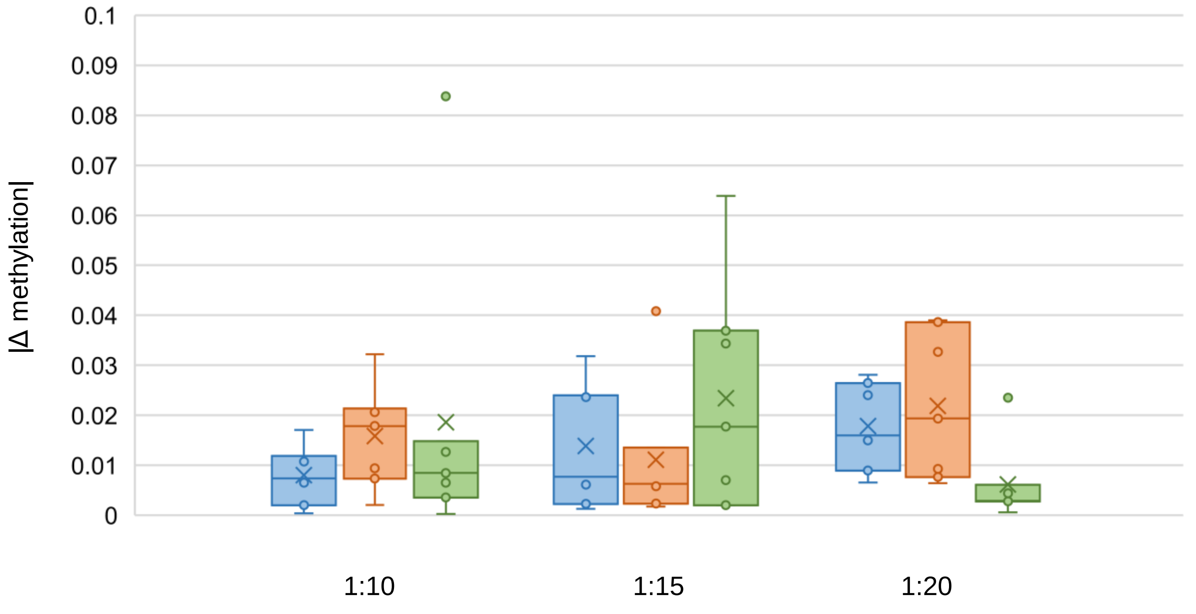

Supplement: Supplementary file 1 — Supplementary file1 (PDF 694 kb) [file 414_2026_3797_MOESM1_ESM.pdf]
